# The association of vitamin deficiency with depression risk in late-life depression: a review

**DOI:** 10.3389/fnut.2025.1551375

**Published:** 2025-04-15

**Authors:** Yao Gao, Xiao-Na Song, Zhong-Ping Wen, Jian-Zhen Hu, Xin-Zhe Du, Ji-Hui Zhang, Sha Liu

**Affiliations:** ^1^Department of Psychiatry, First Clinical Medical College, First Hospital of Shanxi Medical University, Taiyuan, China; ^2^Shanxi Key Laboratory of Artificial Intelligence Assisted Diagnosis and Treatment for Mental Disorders, First Hospital of Shanxi Medical University, Taiyuan, China; ^3^Department of Basic Medical Sciences, Shanxi Medical University, Taiyuan, China; ^4^Department of Mental Health, Sinopharm North Hospital, Baotou, China

**Keywords:** late-life depression, vitamins, clinical efficacy, cellular mechanisms, nutritional intervention

## Abstract

Late-life depression (LLD), a growing public health challenge in aging societies, profoundly impacts physical and mental health by exacerbating cognitive decline, functional disability, and comorbid chronic diseases. Emerging research highlights vitamin supplementation as a promising adjunctive therapy for LLD, targeting its multifactorial pathogenesis involving mitochondrial dysfunction, neuroinflammation, and oxidative stress. Specific vitamins, including B-complex vitamins (B1, B6, B9, B12), vitamin D, and antioxidants (C, E), demonstrate therapeutic potential through mechanisms ranging from neurotransmitter regulation to mitochondrial function enhancement. For instance, vitamin D modulates serotonin synthesis and calcium signaling, while B vitamins mitigate homocysteine-mediated neurotoxicity and support energy metabolism. Antioxidants counteract neural oxidative damage linked to depressive severity. Clinical studies reveal that vitamin D deficiency (<20 ng/mL) correlates with elevated depression risk, and combined B-vitamin supplementation shows symptom alleviation in nutritionally deficient subgroups. However, evidence remains heterogeneous due to variability in dosing protocols, bioavailability, and population-specific factors like comorbidities. Despite growing evidence, critical gaps persist regarding optimal dosages, bioavailability variations, and long-term outcomes in elderly populations. This review synthesizes current evidence on vitamin-mediated cellular pathways in LLD management, evaluates clinical efficacy across interventions, and proposes personalized nutritional strategies to optimize therapeutic outcomes. By integrating mechanistic insights with clinical data, this analysis aims to guide evidence-based vitamin supplementation protocols for LLD within geriatric care frameworks.

## 1 Introduction

Late-life depression (LLD) is a multifaceted disorder influenced by a combination of biological and environmental factors, such as mitochondrial dysfunction, neurotransmitter dysregulation, hormonal imbalances, inflammation, and oxidative stress. As the global aging population continues to grow, the prevalence of depression in older adults is steadily increasing. According to recent studies, the incidence of depression among the elderly is notably higher compared to other age groups, and its symptoms are frequently misattributed to the normal aging process, leading to delayed or inadequate treatment for many patients (1). Depression not only impacts the mental health of the elderly but also elevates the risk of physical health issues, including cardiovascular disease, type 2 diabetes, and Alzheimer’s disease (2, 3). Consequently, identifying and managing depression in later life is of paramount importance.

Recent awareness has grown regarding the potential role of vitamins in enhancing mental health, particularly brain health. Extensive empirical evidence indicates that deficiencies in vitamin D, B vitamins, and other micronutrients are linked to an increased incidence of depression (4, 5), especially among women and overweight older adults (6). Furthermore, evidence suggests that high total intakes of vitamins B6 and B12 exert a protective effect against depressive symptoms in older adults over time (7). Additionally, vitamins B12, D, and E, along with other nutrients, have demonstrated support for mitochondrial function, which is frequently compromised in LLD patients. For instance, niacin supplementation has been shown to increase NAD+ levels and improve mitochondrial function, potentially alleviating depressive symptoms (8). Beyond their role in supporting mitochondrial function, vitamin supplements may also influence inflammation and oxidative stress, both of which are linked to LLD. Vitamin E supplementation, for example, has been found to bolster antioxidant defenses and lower markers of oxidative stress, which are typically elevated in LLD patients (9). By enhancing the body’s capacity to manage oxidative stress and inflammation, vitamin supplements may contribute to improved mental health and cognitive function in older adults. These potential benefits underscore the importance of investigating the integration of vitamin supplements into LLD treatment strategies, offering hope for better outcomes in this population.

In summary, given the growing concerns about aging and associated mental health issues, it is essential to further explore the potential of vitamins as an intervention in future scientific and clinical practices.

## 2 Methods

Search PubMed for vitamins A, B, C, D, E and K and “depression in older adults”; or “LLD”; Or “late-life depression.” Each vitamin is combined with a disease—for example, “vitamin C” or “geriatric depression.” The search is limited to articles published in English within the last 15 years and involving humans. A total of 7,980 articles were retrieved through these searches. Identify relevant papers by reviewing their titles and abstracts; The list of references was also checked to identify any other articles. A total of 28 articles were discussed as they were deemed suitable for inclusion in this review.

## 3 Clinical research and practice

Recent studies have elucidated the multi-dimensional role of vitamin supplements in disease management, yet significant heterogeneity persists in their clinical applications. Research on vitamin D has demonstrated a dose-dependent effect on cancer prevention and metabolic disease regulation; however, consensus regarding its cardiovascular protection remains elusive (10, 11). In the neuropsychiatric domain, vitamin D deficiency (<20 ng/mL) has been robustly linked to an elevated risk of depression in older adults. However, the efficacy of supplemental interventions in alleviating depressive symptoms is moderated by factors such as gender, baseline nutritional status, and others (12–15). The B vitamin family study presents a more intricate scenario: while dietary intake of vitamins B6 and B12 was negatively associated with depression risk, this association was not significant when derived from food sources, underscoring the importance of bioavailability differences (7) (Table 1). Notably, multivitamin interventions have shown synergistic benefits in improving mood and cognitive function in patients with mild cognitive impairment (16, 17), and in combination with n-3 fatty acids, they enhance neuroprotective effects (18, 19). Current evidence underscores the importance of personalized nutrition strategies, including dynamic adjustment of vitamin D dosage based on serum markers (20), balancing the therapeutic window of vitamin B6 against the risk of neurotoxicity (21) and integrating dietary interventions with targeted supplementation to optimize biological outcomes (22–24). These approaches will help improve the effectiveness of vitamin supplementation, mitigate potential side effects, and refine public health strategies and clinical treatment options.

**Table 1:** Functions of vitamins.

Vitamin	Research type	Summary	Reference
Beta-carotene equivalent, vitamin K, vitamin group B, vitamin C	Cross-sectional study	Associations between vitamin deficiencies and depressive symptoms were identified among elderly female participants who are overweight.	(6)
Vitamins B6 and B12	Population-based study; A cross-sectional study	High total intakes of vitamins B6 and B12 are associated with a protective effect against depressive symptoms over time in older adults residing in the community.	(7, 71)
25-hydroxycholecalciferol	A cross-sectional study	Serum 25-hydroxycholecalciferol deficiency was positively associated with depressive symptoms in older adults	(13, 15)
Vitamin B9 and vitamin B12	A randomized controlled trial	Long-term daily oral administration of folic acid and vitamin B12 improved cognitive function, particularly immediate and delayed memory performance	(17)
Carotenoids	Prospective studies	Carotenoid predominance is associated with the risk of depressive symptoms.	(25)
Vitamin B	Cohort study	Better B-vitamin status may have a role in impacting positively on mental health in older adults	(40, 44)
Vitamin B9, vitamin B12 and homocysteine	Cross-sectional and prospective associations	Lower folate, lower vitamin B (12) and raised homocysteine levels may be risk factors for late-life depression	(16, 60)
Vitamin B12 and vitamin B9	The B-PROOF study	Two-year supplementation with vitamin B_12_ and folic acid in older adults with hyperhomocysteinemia showed that lowering Hcy concentrations does not reduce depressive symptoms	(67)
Vitamin C	A randomized, double blind, placebo-controlled trial	A higher proportion of elderly patients have poor vitamin C status, which is associated with increased depressive symptoms	(77)
Vitamin C, beta cryptoxanthin	Cross-sectional association	The importance of food sources of antioxidants in reducing the risk of cardiovascular disease in elderly patients with depression	(78)
Vitamins C and E, carotenoids	Population cohort	Higher intakes of vitamin C, carotenoids, and flavonoids were associated with a lower likelihood of depressive symptoms.	(79)
Vitamin D	Observational studies and randomized trials; A cross-sectional study; A randomized clinical trial	Vitamin D deficiency may be a risk factor for geriatric depression. Vitamin D supplementation can improve depression scores in the elderly aged 60 years and older.	(12, 80–82, 92)
Vitamin K		High intake of vitamin K may reduce the risk of depression	(117)

## 4 Role of vitamins in depression

### 4.1 Vitamin A

**Figure 1:**
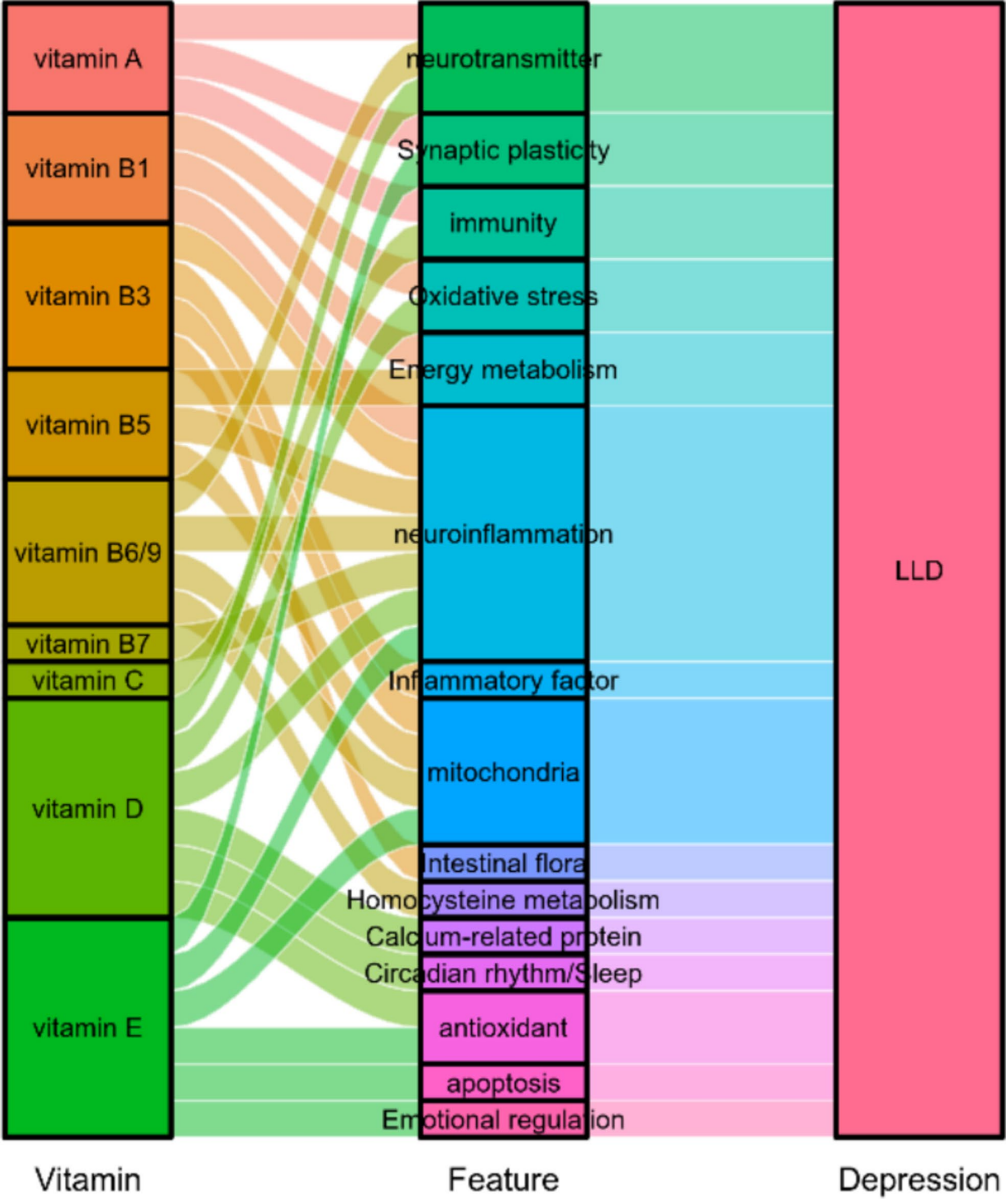
The potential role of vitamins in late-life depression.

Carotenoids are a class of fat-soluble pigments with antioxidant, immunomodulatory, and neuroprotective properties. They can be categorized into carotenes (such as *α*-carotene, *β*-carotene, *γ*-carotene, and lycopene) and xanthophylls (such as lutein, zeaxanthin, and β-cryptoxanthin) based on their chemical structure. Notably, α-carotene, *β*-carotene, γ-carotene, and β-cryptoxanthin function as provitamin A compounds, which can be metabolized to form vitamin A. Research has demonstrated that elevated plasma levels of zeaxanthin and *β*-carotene are significantly associated with a reduced risk of depression in older adults (25). Additionally, total carotenoid and β-carotene concentrations have been found to be negatively correlated with temporal lobe atrophy, indicating a potential protective effect against age-related neurodegeneration (26). Importantly, while skin carotene content is positively correlated with information processing speed, it does not appear to be associated with attention or executive function, suggesting its potential as a specific biomarker for cognitive decline. Retinol, a precursor to vitamin A, and retinoic acid (RA), a metabolite in the nervous system, play crucial roles by regulating synaptic plasticity (27) and neurotransmitter homeostasis. Differences in retinol receptor distribution in the adult human brain (28, 29) suggest its involvement in the functional regulation of emotion-related brain areas such as the hippocampus and amygdala. Further research has demonstrated that RA signaling is enriched in depression-related brain regions (30). The synaptic scaling effect mediated by RA (31) and homomorphic regulation mediated by the RARα receptor (32–34) may rapidly exert antidepressant effects by restoring neurotransmitter balance. While vitamins exhibit potential in the biological mechanisms and treatment strategies for LLD (Figure 1), concerns regarding species differences, dose-dependent effects, and potential toxicity risks must be addressed. Future studies should aim to establish more precise associations between their neuroregulatory pathways and clinical translational feasibility.

### 4.2 Role of B vitamins in depression

#### 4.2.1 Vitamin B group

As a family of water-soluble coenzymes, B vitamins play a central role in maintaining nervous system health by synergistically regulating key pathways of energy metabolism. Specifically, thiamine (Vitamin B1) catalyzes the oxidative decarboxylation reactions in the citric acid cycle, niacin (B3) facilitates oxidative phosphorylation via its role as NADH, pyridoxine (Vitamin B6) is crucial for amino acid metabolism, neurotransmitter synthesis, and lipid metabolism (35). Pantothenic acid (Vitamin B5) and biotin (Vitamin B7) function as coenzyme A and carboxylase cofactors, respectively, participating in the tricarboxylic acid cycle and fatty acid oxidation. Folic acid (Vitamin B9), in its reduced form as tetrahydrofolate, functions as an important cofactor in methylation reactions and as a carrier of carbon units in the synthesis of purines and pyrimidines (36, 37). This multi-target regulatory property makes B vitamins ideal candidates for addressing energy metabolism disturbances in LLD. Studies have demonstrated that LLD is closely associated with energy metabolism disorders, potentially stemming from impaired mitochondrial function and chronic inflammation, leading to neuronal apoptosis and oxidative stress (38). The brain, being an energy-intensive organ, relies heavily on ATP homeostasis maintained by B vitamins for processes such as synaptic transmission and neuroplasticity (39). Clinical evidence indicates that low levels of vitamin B1, B5, B6, and B9 are prevalent among LLD patients, with their deficiencies significantly correlated with the severity of depression and the rate of cognitive decline (6). Studies have shown that regular intake of fortified foods can optimize B vitamin status and thus help reduce depression (40) (Table 1). These findings underscore the multiple mechanisms of action and clinical application prospects of B vitamins in managing LLD. Restoring physiological levels of B vitamins through dietary supplementation or targeted interventions may offer new strategies to improve neuronal energy metabolism imbalance.

#### 4.2.2 Vitamin B1: thiamine

Vitamin B1, as a central regulator of energy metabolism, directly facilitates mitochondrial ATP production by catalyzing the activities of *α*-ketoglutarate dehydrogenase and pyruvate dehydrogenase complexes in the tricarboxylic acid (TCA) cycle through its active form, thiamine pyrophosphate (TPP) (41). In LLD, vitamin B1 deficiency can impair TCA cycle function, leading to disrupted energy metabolism in emotion-regulating brain regions such as the hippocampus and prefrontal cortex (37). This disruption may be a critical mechanism underlying fatigue and cognitive impairment in LLD patients. Clinical studies have demonstrated that thiamine supplementation enhances the activity of enzymes related to mitochondrial function (42–44) and significantly alleviates depressive symptoms (45). Notably, the neuroprotective effects of vitamin B1 extend beyond supporting energy metabolism; it also effectively mitigates neuroinflammation and oxidative stress by inhibiting the release of pro-inflammatory cytokines such as TNF-*α*, IL-1β, and IL-6 (46–48). Observational studies further indicate that maintaining adequate levels of vitamin B1 is positively correlated with cognitive function, including attention, working memory, and emotional regulation (49, 50) (Figure 1). These findings suggest that vitamin B1 supplementation may offer potential therapeutic strategies for improving brain function in LLD patients by synergistically regulating energy metabolism and inflammatory responses.

#### 4.2.3 Vitamin B3: niacin

Vitamin B3, also known as niacin, plays a critical role in energy metabolism and mitochondrial function, which is essential in the context of LLD. Vitamin B3 serves as a precursor to nicotinamide adenine dinucleotide (NAD+), a coenzyme vital for cellular redox reactions and energy production. Research by Lapatto et al. indicates that vitamin B3, specifically niacinamide riboside, may enhance muscle mitochondrial function and modulate gut microbiome composition in humans (51). Furthermore, studies have shown that vitamin B3 can reduce oxidative stress and neuroinflammation, both of which are closely linked to the pathogenesis of LLD (52). By improving mitochondrial function and mitigating oxidative damage, vitamin B3 may offer a therapeutic avenue for alleviating LLD symptoms and enhancing overall brain health in older adults. This underscores its potential as a supportive treatment and addresses the interplay between metabolic and inflammatory aspects of the disease. Additionally, Liu et al. suggest that vitamin B3’s modulation of ATP, independent of SIRT1 activity, could be a promising approach for treating depressive disorders (53). Consequently, vitamin B3 may serve as a beneficial intervention for depression in elderly patients with LLD.

#### 4.2.4 Vitamin B5: pantothenic acid

Vitamin B5, also known as pantothenic acid, plays a crucial role in energy metabolism as it serves as a precursor to the synthesis of Coenzyme A. Coenzyme A is an essential cofactor in numerous biochemical reactions, particularly those involved in energy production and mitochondrial function (54). The significant connection between vitamin B5 and energy metabolism implies its potential to influence the pathophysiological processes of LLD by enhancing mitochondrial health and boosting energy production. Research has demonstrated that vitamin B5 facilitates mitochondrial respiration and metabolic maturation, and exerts a critical regulatory effect on intracellular energy homeostasis (55). Given that mitochondrial dysfunction is linked to LLD, the role of vitamin B5 in supporting mitochondrial function may offer a therapeutic avenue to alleviate depressive symptoms in older adults. Furthermore, vitamin B5 is implicated in the regulation of inflammation, a critical factor in the onset and progression of LLD. Chronic inflammation can induce neuronal apoptosis and oxidative stress, thereby exacerbating depression in the elderly (38). Vitamin B5 exerts its anti-inflammatory effects by decreasing levels of inflammatory mediators and facilitating a shift towards an anti-inflammatory pathway (56). This anti-inflammatory potential enables vitamin B5 to mitigate immune disorders associated with LLD and improve depressive symptoms and overall prognosis. By modulating energy metabolism and inflammation, vitamin B5 may play multifaceted roles in addressing the underlying biological mechanisms of LLD, offering a promising adjunctive treatment strategy.

#### 4.2.5 Vitamin B7: biotin

Vitamin B7, also known as biotin, has been identified as a potential modulator of neuroinflammation and neurotransmitters, both of which are implicated in LLD. Reininghaus et al. demonstrated that a 4-week regimen of probiotics combined with biotin supplementation in patients with major depression resulted in significant clinical benefits (57). Biotin has been shown to ameliorate neuroinflammatory dysfunctions, and in animal models, a novel biotin complex called bionicotinate magnesium (MgB) improved social behavior, learning, and memory deficits (58). These findings suggest that vitamin B7 may help mitigate the inflammatory processes associated with LLD. Furthermore, the combination of manganese chloride and biotin has been found to upregulate brain CYP1B1 expression and reduce neurotoxicity, indicating a protective effect on neurotransmitter regulation and cerebrovascular homeostasis (59). These results indicate that vitamin B7 may support neurotransmitter balance, reduce neuroinflammatory responses, and thereby alleviate some symptoms of LLD. Although further scientific evidence is required to confirm the therapeutic potential of vitamin B7 in LLD, the preliminary findings highlight its promising role in addressing the complex physiological mechanisms underlying this condition.

#### 4.2.6 Vitamin B6/9/12

LLD is a complex condition influenced by the interplay between biological and environmental factors. Its pathophysiological mechanisms involve homocysteine metabolism disorders, mitochondrial dysfunction, and neuroinflammatory cascades. Research has demonstrated that hyperhomocysteinemia can exacerbate depressive symptoms in elderly individuals by inducing neurovascular damage and oxidative stress (60–62). The methylation metabolic network, which includes vitamins B6, B9, and B12, plays a central regulatory role in this process. Specifically, vitamin B6 acts as a cofactor for cystathionine *β*-synthase, facilitating the conversion of homocysteine to cysteine (63, 64). Vitamin B12, through methionine synthase, remethylates homocysteine to methionine, influencing the synthesis of neurotransmitters such as dopamine and norepinephrine (65). Clinical evidence indicates that combined supplementation of B9 and B12 can significantly reduce elevated homocysteine levels (66); however, its effect on depressive symptoms remains controversial. For instance, De Koning et al. found no antidepressant effect during a 2-year intervention (67), while Skarupski et al. observed that higher dietary intake of B6 and B12 was associated with reduced depressive symptoms (7). A very recent study showed that the current minimum recommendations were not designed for optimal cognitive function or longevity as participants with B12 levels deemed adequate by today‘s medical standards showed clear signs of neurological impairment, while healthy older adults (averaging 71 years of age) who did not have dementia or mild cognitive impairment averaged B12 levels at 414.8 pmol/L, much higher than the recommended minimum level (68). This inconsistency may be attributed to differences in study design, such as intervention duration or baseline nutritional status, or brain region-specific metabolic responses. Importantly, the role of B vitamins extends beyond homocysteine regulation. Vitamin B6 targets neurotransmitter imbalances and neuroinflammation by participating in 5-hydroxytryptamine synthesis (69, 70) and inhibiting the NF-κB pathway (9, 56). Cohort studies have further demonstrated that insufficient vitamin B6 intake is positively correlated with increased depression severity in middle-aged and older women (71). Additionally, deficiencies in both vitamins B6 and B9 are dose-dependently associated with cognitive decline and changes in brain structure (72, 73). While existing evidence underscores the multi-target potential of B vitamins in preventing and treating LLD, it remains essential to establish a therapeutic window and biomarker-guided personalized application strategies through standardized intervention trials.

### 4.3 Vitamin C

Vitamin C (ascorbic acid), a crucial water-soluble antioxidant, exerts multidimensional protective effects on the health of the elderly by scavenging free radicals, promoting collagen synthesis, and modulating immune function. Research has demonstrated that short-term vitamin C supplementation can significantly enhance immune function in older adults (74). Moreover, dietary intake of vitamin C is inversely associated with the incidence of depression, particularly among elderly women (75, 76). Clinical observations further indicate that vitamin C deficiency is not only strongly linked to increased depressive symptoms following acute illness (77) but may also accelerate cognitive decline and increase susceptibility to infections. Notably, inadequate vitamin C intake is prevalent among the elderly globally, especially among those with limited access to fresh fruits and vegetables (78, 79). Therefore, implementing vitamin C fortification interventions for high-risk groups could serve as an important public health strategy to improve the quality of life of the elderly and reduce disease burden.

### 4.4 Vitamin D

Vitamin D is a fat-soluble vitamin, with its metabolically active form, 1,25-dihydroxyvitamin D3 (1,25(OH)2D3), exerting neuroprotective effects via the vitamin D receptor (VDR), which is widely distributed in the cerebral cortex, cerebellum, and substantia nigra. Research has demonstrated a significant association between vitamin D deficiency and the occurrence of depressive symptoms in elderly individuals, particularly during seasons with low sunlight exposure (80, 81) (Table 1). Longitudinal studies have further confirmed that fluctuations in vitamin D levels can predict the development of depressive symptoms (12, 82). The antidepressant mechanisms of vitamin D involve multi-pathway regulation: maintaining neuronal function by regulating calcium ion homeostasis-associated proteins (83–85); enhancing the expression of tryptophan hydroxylase 2 (TPH2) through VDR activation, thereby promoting serotonin synthesis and inhibiting its metabolism to enhance monoaminergic neurotransmission (86–89); upregulating antioxidant genes such as NRF2 and inhibiting the inflammatory pathway of NF-κB, thus alleviating nerve oxidative damage (90, 91). Additionally, vitamin D influences circadian rhythms by modulating the serotonin-melatonin conversion axis and exerts potential protective effects through interactions with sex hormones (92). Clinical intervention studies have shown that supplementation with vitamin D3, combined with vitamins B6 and K1, effectively alleviates oxidative stress (93), and meta-analyses support its potential to improve depressive symptoms (94). However, other studies have shown that neither vitamin D3 nor omega-3 has shown any benefit for targeted and selective prevention of depression in the elderly (95). It is noteworthy that although vitamin D is one of the least toxic vitamins, excessive intake can lead to adverse clinical symptoms such as confusion, apathy, recurrent vomiting, and abdominal pain (96). Therefore, vitamin D intake should be maintained within a reasonable range. In summary, vitamin D deficiency is a risk factor for exacerbating depression in old age by interfering with neurotransmitter balance, calcium signaling, and antioxidant defense systems (Figure 1).

### 4.5 Vitamin E

Vitamin E is a group of eight vitamers, four tocopherols and four tocotrienols, and both occur in alpha, beta, gamma, and delta forms. It exerts its neuroprotective effects through a multifaceted regulatory mechanism: by scavenging free radicals, it alleviates oxidative stress damage; additionally, it enhances mitochondrial energy metabolism efficiency (97, 98); modulates signaling pathways associated with synaptic plasticity, thereby influencing emotional and cognitive functions (99); and inhibits inflammatory pathways such as NF-κB, reducing neuroinflammatory responses mediated by microglia activation (100–102). Preclinical studies have confirmed that vitamin E supplementation significantly mitigates anxiety-like behaviors (103, 104) and reverses age-related immune disorders (105–107). Vitamin E plays a protective role in the pathological process of LLD via two mechanisms: first, it suppresses the expression of apoptosis-related molecules such as caspase-3, preserving neuronal structural integrity; second, it promotes hippocampal neurogenesis and repairs emotion-related neural circuits (108, 109). Observational studies further revealed a significant negative association between combined dietary intake of vitamins E and C and the incidence of depression (110). Collectively, these findings suggest that vitamin E may be a key nutrient in multi-target intervention strategies for LLD by synergistically regulating REDOX balance, inflammatory cascades, and apoptosis processes (Figure 1).

### 4.6 Vitamin K

As a neuroprotective, fat-soluble vitamin, vitamin K in its active form methylnaphthoquinone-4 (MK-4) performs multiple biological functions in the central nervous system by participating in sphingoid metabolism. These functions include promoting nerve growth factor expression, regulating myelin formation, and maintaining neuronal homeostasis (111). Epidemiological studies have demonstrated that vitamin K intake is significantly negatively correlated with depressive symptoms (112). The underlying mechanisms involve multi-pathway synergistic regulation: down-regulating pro-inflammatory factors such as IL-1β and IL-6 while up-regulating IL-10 (113), thereby blocking the neuroinflammatory cascade (114); inhibiting lipid peroxidation and ferroptosis to maintain redox balance (115); activating the Sirt1-PGC-1α-TFAM signaling axis to enhance mitochondrial biogenesis and improve energy metabolism disorders (116). Notably, vitamin K2 alleviates LLD-related cognitive impairment and mood disorders by regulating mitochondrial autophagy and neurotransmitter homeostasis, particularly within the serotonin system (65). Although the direct causal relationship between vitamin K and LLD remains to be confirmed, existing evidence suggests that targeting the “inflammatory-oxidative-mitochondrial” pathological axis offers new insights for precision nutritional interventions for depression in older adults (6, 115, 117).

## 5 Conclusion

Vitamin supplements have shown multi-target intervention potential in managing LLD by modulating biological mechanisms such as neurotransmitter metabolism, inflammatory pathways, and oxidative stress. Existing studies have confirmed that specific nutrients, including vitamin D, B complex vitamins, and vitamin C, can alleviate mood disorders through enhancing neuroplasticity, regulating homocysteine metabolism, and scavenging free radicals, respectively. However, the heterogeneity of clinical trial results, particularly dose-dependent variations in vitamin D efficacy, underscores the need for cautious interpretation of available evidence. Standardization of study design, including baseline nutritional status assessment and controlled intervention cycles, along with population stratification based on chronic comorbidities, will be crucial to overcoming current limitations. Future research should focus on three key dimensions: (1) Establishing quantitative models linking nutrients, biomarkers, and clinical symptoms; (2) Investigating the synergistic effects of vitamin supplementation combined with psychological or pharmacological interventions; and (3) Developing personalized supplementation strategies based on metabolomics characteristics. These advancements will facilitate the transition from empirical supplementation to precise intervention in nutritional psychiatry, providing a theoretical foundation for a multi-modal treatment system for geriatric depression.
